# Enzymatic Synthesis of Highly Fluorescent 8-Azapurine Ribosides Using a Purine Nucleoside Phosphorylase Reverse Reaction: Variable Ribosylation Sites

**DOI:** 10.3390/molecules181012587

**Published:** 2013-10-11

**Authors:** Alicja Stachelska-Wierzchowska, Jacek Wierzchowski, Beata Wielgus-Kutrowska, Goran Mikleušević

**Affiliations:** 1 Department of Biophysics, University of Varmia & Masuria, 4 Oczapowskiego St., 10-719 Olsztyn, Poland; E-Mail: jacek.wie@uwm.edu.pl; 2 Division of Biophysics, Institute of Experimental Physics, University of Warsaw, Zwirki i Wigury 93, 02-089 Warsaw, Poland; E-Mail: beata@biogeo.uw.edu.pl; 3 Division of Physical Chemistry, Rudjer Bošković Institute, POB 180, HR-10002 Zagreb, Croatia; E-Mail: Goran.Mikleusevic@irb.hr

**Keywords:** 8-azapurines, nucleosides, enzymatic synthesis, fluorescence, purine-nucleoside phosphorylase

## Abstract

Various forms of purine-nucleoside phosphorylase (PNP) were used as catalysts of enzymatic ribosylation of selected fluorescent 8-azapurines. It was found that the recombinant calf PNP catalyzes ribosylation of 2,6-diamino-8-azapurine in a phosphate-free medium, with ribose-1-phosphate as ribose donor, but the ribosylation site is predominantly N7 and N8, with the proportion of N8/N7 ribosylated products markedly dependent on the reaction conditions. Both products are fluorescent. Application of the *E. coli* PNP gave a mixture of N8 and N9-substituted ribosides. Fluorescence of the ribosylated 2,6-diamino-8-azapurine has been briefly characterized. The highest quantum yield, ~0.9, was obtained for N9-β-d-riboside (λ_max_ 365 nm), while for N8-β-d-riboside, emitting at ~430 nm, the fluorescence quantum yield was found to be close to 0.4. Ribosylation of 8-azaguanine with calf PNP as a catalyst goes exclusively to N9. By contrast, the *E. coli* PNP ribosylates 8-azaGua predominantly at N9, with minor, but highly fluorescent products ribosylated at N8/N7.

## 1. Introduction

**Figure 1:**
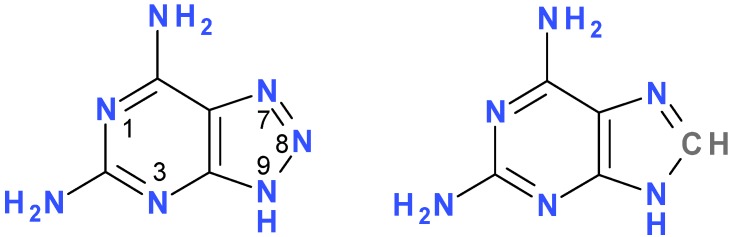
Structures of 2,6-diamino-8-azapurine (left) and 2,6-diaminopurine. The compounds are shown in one of several possible tautomeric forms. Purine numbering is maintained in both systems for simplicity.

Enzymatic ribosylation of purine analogues has been proposed as an alternative to chemical synthesis of a variety of biologically active nucleosides [1,2,3,4]. We have been interested in fluorescent analogues of natural purine nucleosides and nucleotides, since they have found numerous applications in enzymology, particularly in mechanistic and dynamic studies (for reviews, see [5,6,7]). Among these, 8-azapurine nucleosides are known as fluorescent species, isosteric with natural purine nucleosides, and may substitute for them in many biochemical processes [8,9]. They are suitable for mechanistic investigations, including probing of the active sites of purine-related enzymes and ribozymes [10,11,12,13,14], as well as for analytical purposes [15,16]. In the present work, we examined a possibility of enzymatic synthesis of ribosides of 2,6-diamino-8-azapurine (Figure 1), a highly fluorescent purine analog [17], using as catalysts various forms of purine-nucleoside phosphorylase (PNP, for a review see [18]), particularly trimeric calf PNP and hexameric *E. coli* PNP, and α-d-ribose-1-phosphate (R1P) as a ribose donor. Ribosides and deoxyribosides of 2-amino-8-azaadenine have been long ago synthesized by Montgomery [19] and Seela and Lampe [20], but their fluorescence properties were not described.

The parent purine nucleoside, 2,6-diaminopurine riboside, is known to possess interesting biochemical properties [21,22,23] and was recently applied to mechanistic studies of the hairpin ribozyme [24], which expressed full activity when the crucial guanine residue was replaced by 2,6-diaminopurine (Figure 1). However, 2,6-diaminopurine and its nucleoside are only moderately fluorescent [21], and therefore are not easily applicable to fluorescent investigations in biological systems. By contrast, the 2,6-diamino-8-azapurine (2-amino-8-azaadenine) ribosides and related compounds exhibit very intense fluorescence in neutral aqueous media, as shown below. 

## 2. Results and Discussion

### 2.1. Fluorescence of 2,6-diamino-8-azapurine Base and its Alkyl Derivatives

**Figure 2:**
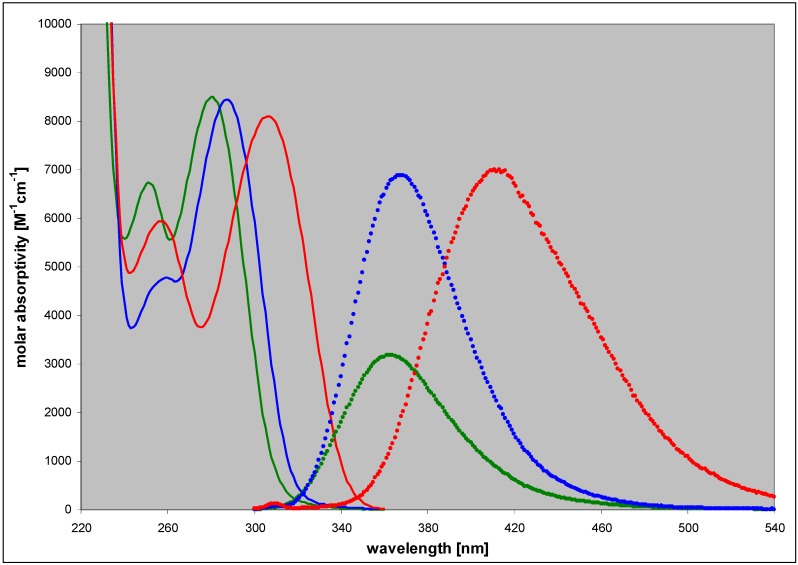
Absorption (left, solid lines) and emission (right, points) spectra of the neutral forms of DaaPur (green), N9-propoxyphosphono-DaaPur (blue) and N8-methyl-DaaPur (red) in aqueous medium, pH 6–7 (Data from [17]). Fluorescence spectra were normalized to the lowest-energy UV absorption bands and multiplied by the respective quantum yields.

In contrast to the parent, weakly fluorescent 2,6-diaminopurine, its 8-aza analogue (2,6-diamino-8-azapurine, DaaPur), exhibits strong fluorescence in neutral aqueous medium, with a maximum at 365 nm and 40% yield, described in details in our previous paper [17]. This fluorescence was ascribed to the N(9)H protomer, on the basis of comparison with the emission properties of N9-alkoxyphosphono-derivative of DaaPur (see Figure 1). Other N-alkyl derivatives, N8-methyl and N7-methyl-DaaPur, are characterized by red-shifted UV absorption [25], and N8-methyl-DaaPur additionally exhibits intense fluorescence centered at 410 nm (Figure 2). On the basis of the foregoing observations, we expected that ribosides of DaaPur may exhibit favorable emission properties, useful for analytical and structural studies. 

### 2.2. Enzymatic Ribosylation of DaaPur Using Various Forms of PNP as a Catalyst

**Figure 3:**
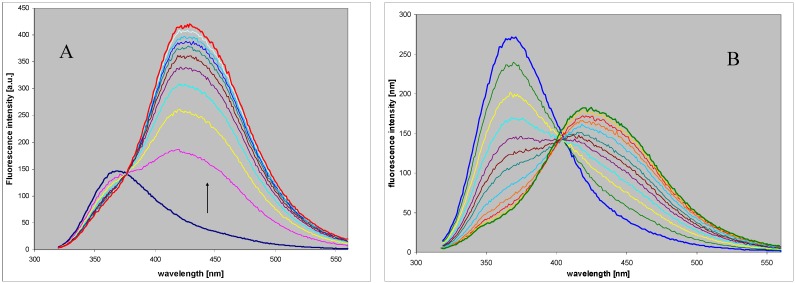
Fluorescence changes during the enzymatic ribosylation of DaaPur (~10 µM) with ribose-1-phosphate (0.25–0.5 mM), using recombinant *E. coli* (left) and calf spleen (right) purine-nucleoside phosphorylase as catalysts. Reactions were carried out in 25 mM HEPES, pH 6.6, at 25 °C, for ~1.5 h. Spectra were measured every 5 min for 30 min and every 20 min afterwards. Excitation wavelength was at 310 nm.

Although 2,6-diaminopurine, like adenine, is not a substrate for the mammalian forms of PNP in the reverse (synthetic) pathway [18], its 8-aza analog, DaaPur, has been found to be a modest substrate for both calf and *E. coli* PNP’s (wild types), with either ribose-1-phosphate or N7-methylguanosine as ribose donors. Reaction progress can be followed using UV and/or fluorescence spectroscopy, as shown in Figure 3 for the reaction between ribose-1-phosphate and DaaPur. The reaction rate for the calf enzyme at pH 6.6 is about 5% that of the 8-azaGua and ~0.4% of Gua ribosylation (see Table 1). For the *E. coli* PNP, ribosylation of DaaPur runs at ~2% of the ribosylation of guanine. 

**Table 1:** Kinetic parameters for the enzymatic ribosylation of purine analogues with various form of PNP as catalysts, and α-D-ribose-1-phosphate as a co-substrate. Data refer to pH 6.5.

Compound	Relative ^a^ k_cat_	K_m_ [µM]	Enzyme source
2,6-diamino-8-azapurine	~0.4%	54	calf spleen
	2%	65	*E. coli*
2,6-diaminopurine ^b^	<0.1%	-	calf spleen
	>120% ^c^	~7	*E. coli*
8-azaguanine ^c^	21%	100	calf spleen
	~4 U/mg	>200	*E. coli*

^a^ relative to those determined for guanine (=100%); ^b^ data from [15], at pH 7; ^c^ relative to the parameters determined for N7-methylguanosine phosphorolysis at pH 6.5 (=100).

It is evident from the spectra recorded during the reaction course that in both cases the ribosylation product(s) involve species absorbing at ~310 nm and strongly emitting near 430 nm, thus markedly differing from the substrate. These products must be distinct from the typical N9-riboside, and spectrally resembling the N8-alkyl derivative of DaaPur (cf. Figure 2 and Figure 3). At the endpoint of the reaction catalyzed by calf PNP (Figure 2B) the short-wavelength band at 365 nm virtually disappears, suggesting that little or no N9-riboside is produced. Occasionally, a lack of the isoemissive point was observed (see Figure 2), indicating existence of more than one product, likely N8- and N7-ribosides, confirmed by the HPLC analysis (see below). In the case of the *E. coli* PNP, the reaction mixture contained a marked component of the N9-riboside as well, emitting at ~365 nm, further documented below.

**Figure 4:**
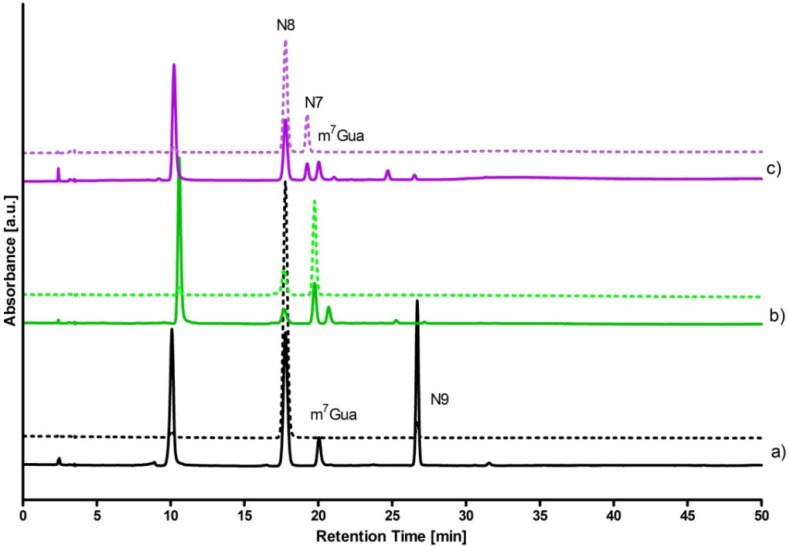
Elution profiles for the HPLC analysis of the enzymatic ribosylation of DaaPur, catalyzed by: (**a**) *E. coli* PNP; (**b**) calf spleen PNP, 4 h reaction in the absence of phosphate; (**c**) calf spleen PNP, 8 h reaction in the presence of 0.5 mM phosphate. Detection was at 280 nm (solid line) and 315 nm (broken line). Peak near 10 min retention time is the substrate.

We have separated the ribosylation products using HPLC, with C-8 column (Kromasil) in the reverse-phase mode, with diode array absorption detector (see Experimental section for details). The elution profiles, given in Figure 4, confirm that the ribosylation of DaaPur with calf PNP as a catalyst gives two main products, with UV absorption spectra extending much >300 nm. 

**Table 2:** Ionization constants (pK_a_ values) and spectral parameters for 2,6-diamino-8-azapurine, its ribosides and selected derivatives. The UV and fluorescence spectral data for DaaPur and derivatives are from [8,17,19,20,25], additionally checked in the present work.

Compound	p*K_a_*	Form (pH) ^a^	UV absorption ^b^		Fluorescence	
			λ_max_ [nm]	ε_max_ [M^−1^cm^−1^]	λ_max_ [nm]	ϕ
2,6-diamino-8-azapurine	3.68; 7.68	n (6)	280	8500	363	0.40
		ma (10)	290	6400	370	0.36
		cat (2.5)	253	9500	410	0.27
N9-phoshonomethoxy-propyl(PMP)-	3.4	n (7)	284	nd ^c^	367	0.80
N8-methyl-	4.85	n (7)	307	8100	412	0.85
		cat (2.5)	284	12000	410	~0.4
N7-methyl-	4.3	n (7)	309	6200	nd	nd
		cat (2)	282	7900	nd	nd
N9-β-D-ribofuranosyl-	~3.2	n (7)	286	10000	367	~0.9
N8-β-D-ribofuranosyl-	4.9	n (7)	313	8200	430	0.41
N7-β-D-ribofuranosyl-	4.1	n (7)	314	~5500	420	0.06

^a^ n—neutral form; cat—cation; ma—monoanion; ^b^ molar absorptivities for the ribosides has been determined relative to that of DaaPur, following enzymatic phosphorolysis; ^c^ nd—not determined.

Both products are fluorescent, with emission maxima at 420–430 nm (see Table 2). The products, differing by the protonation constants (pK_a_), were identified as N8- and N7-ribosides by comparing spectral data with those published in refs [19,20], and with data reported for the alkyl derivatives of DaaPur [17].

By contrast, using *E. coli* PNP as catalyst leads to production of two well separated and spectrally distinct products (Figure 3), one of which was identified as N8-riboside, identical to that produced by the calf PNP, and the second as N9-riboside, which was also highly fluorescent, but with maximum close to 367 nm, very similar to the N9-alkylated DaaPur (Table 2). 

It is interesting that the relative proportion of the products in both cases depends markedly on the reaction conditions, and particularly on the presence of free inorganic phosphate. When no phosphate was present initially, the main products were N7-riboside and N8-riboside for the reaction catalyzed by the calf and *E. coli* PNP, respectively. But when the reaction was started in the presence 0.5 mM phosphate, or if the reaction was allowed to proceed for 24 h, the apparent trans-ribosylation occurred, resulting in much higher proportion of the N8-riboside in the case of calf PNP catalysis (see Figure 4), and of N9-riboside in the reaction catalyzed by the *E. coli* enzyme (data not shown). This phenomenon is of interest not only from the mechanistic point of view, but also as a tool of selective synthesis of ribosides of DaaPur.

### 2.3. Spectral and Biochemical Properties of the Ribosylation Products

Spectral parameters of the purified ribosides, compared to those of DaaPur and its alkyl derivatives are summarized in Table 2. It is evident that N9-β-d-ribofuranosyl-DaaPur and N8-β-d-ribofuranosyl-DaaPur are the most intensely fluorescent nucleosides, comparable to 2-aminopurine riboside (ϕ ~ 0.6 [21]), and 1,N^6^-ethenoadenosine (ϕ = 0.55 [26]). Since the N9-riboside is isosteric with natural purine nucleosides, it is particularly suitable for biophysical applications. It is also interesting that in acidic media both N8- and N7-riboside reveal fluorescence spectra characteristic for the neutral forms (data not shown), which is due to rapid deprotonation of the excited cations (to be reported elsewhere).

We have also examined substrate properties of the DaaPur ribosides towards PNP in the presence of inorganic phosphate (phosphorolytic pathway). The N9-β-d-ribofuranosyl of DaaPur is not a substrate for the calf enzyme, similarly to the analogous riboside of 2,6-diaminopurine [18]. Only traces of activity were recorded for N8-β-d-ribofuranosyl-DaaPur. By contrast, the N7-isomer was phosphorolysed with the rate constant (k_cat_/K_m_) ca. 2% that of N7-methyguanosine phosphorolysis. All three ribosides were phosphorolysed by the *E. coli* enzyme, and the greatest rate constant was recorded for the N8-riboside [16]. The fluorogenic effect observed during the phosphorolysis of N7- and N8-ribosides may be analytically useful, as reported elsewhere [16]. 

### 2.4. Comparison with Enzymatic Ribosylation of 8-azaguanine and Other 8-azapurines

**Figure 5:**
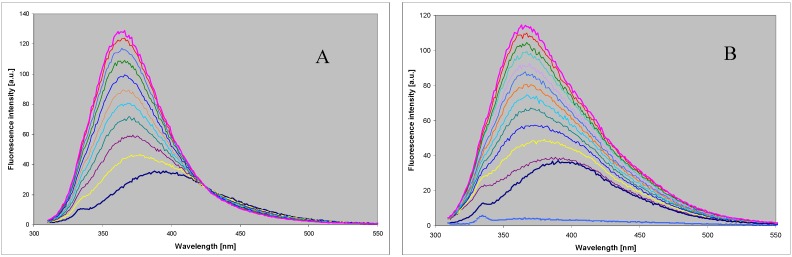
Fluorescence changes recorded during the enzymatic ribosylation of 8-azaGua (~10 µM) catalyzed by the recombinant purine nucleoside phosphorylase from calf (left panel) or *E. coli* (right panel) PNP, with ribose-1-phosphate as a ribose donor. Reactions were carried out in 20 mM HEPES, pH 7.3 at 25 °C. Excitation was at 300 nm.

As previously reported [9], 8-azagauanine (8-azaGua) is effectively ribosylated using calf PNP in phosphate-free media, at pH ≤ 8, with either R1P or N7-methylguanosine as ribose donors. The *E. coli* PNP is a less effective catalyst, with k_cat_ only of ~1% that for guanosine synthesis, while for the calf PNP this value is increased to ~20%. Until now, we have assumed, mainly on the basis of UV absorption spectral changes, that in both cases the only ribosylation product is 8-azaguanosine (N9-β-d-8-azaguanine riboside). The unexpected findings obtained for DaaPur ribosylation prompted us to re-examine the ribosylation of 8-azaGua, using fluorescence spectroscopy and HPLC, with results shown on Figure 5. The reaction was carried out at pH 7.3, which was not optimal from the kinetic point of view [9], but allowed sensitive detection of the product(s) when excitation wavelength is set at 300 nm. 

Initially, the substrate (8-azaGua) is mostly in the anionic form (pK_a_ 6.5), which is weakly fluorescent in these conditions and the observed fluorescence band with λ_max_ 395 nm is due mostly to the small fraction of the neutral form present [9]. During the reaction course (Figure 5), a new band with a maximum at 360 nm appears, ascribed to the strongly fluorescent anionic form of the N9-β-D-riboside (pK_a_ 8.0). With the reaction catalyzed by calf PNP, this is the only fluorescent product formed, since at the reaction endpoint the fluorescence spectrum of the final mixture is independent of excitation wavelength (data not shown). By contrast, when the *E. coli* PNP was applied as catalyst, the appearance of the second, minor fluorescent product was evident, since the resultant fluorescence was red-shifted (Figure 5B) and excitation-dependent, with emission maximum shifting to ~415 nm with excitation at 315 nm (data not shown). The long-wavelength fluorescence is most likely due to N8- and/or N7-ribosides.

The foregoing observations were confirmed by HPLC analysis (data not shown). The reaction catalyzed by *E. coli* PNP gave clearly two nucleoside products, with spectral properties corresponding to N9- (major product, λ_max_ = 256 nm) and N8-riboside (minor, highly fluorescent product). The proportion of two products was nearly 20:1. For the reaction catalyzed by the calf PNP, the minor peak was absent.

The ribosylation site ambiguity described here for DaaPur has also been observed for 8-azaadenine and 8-azaisoguanine, when these were ribosylated using *E. coli* PNP (unpublished data), as well as for 8-azaxanthine, when ribosylated in similar conditions using xanthosine phosphorylase from *E. coli* as a catalyst [27]. It should be recalled, at this point, that the non-typical N7-β-D- and N3-β-D-ribosides of natural purine bases are moderate to good substrates for both mammalian and bacterial enzymes in the phosphorolytic pathway [28,29], so the presence of these ribosides in the reverse reaction is not as surprising, as is perhaps their absence in the reactions catalyzed by the mammalian enzymes.

The absence of N9-riboside product during the ribosylation of DaaPur with the calf PNP is understandable in view of the general properties of mammalian PNP’s, which for the effective catalysis require interaction between purine N(1)-H, which is absent in DaaPur, and the essential glutamate residue of the enzyme (Glu-201 in the calf PNP) [18]. This interaction is not necessary for the *E. coli* PNP, as well in some mutants of the calf enzyme. It is difficult to explain the mechanism of the ribosylation at N7, not observed for the parent 2,6-diaminopurine. One possibility is binding of the 8-azapurine substrate turned by 180 degrees around the long axis, with amine group interacting with Glu201 and N9 replacing N7 in interaction with Asn243 (cf. ref. [18]).

The slow trans-ribosylation of DaaPur ribosides from N7 to N8 position, and from N8 to N9, observed for the reactions catalyzed by the calf and *E. coli* PNP, respectively, is important for synthetic applications, allowing selective synthesis of the required ribosides. Its mechanism is not clear, and probably different than that used by Mikhailopulo *et al*. [30] for synthesis of nucleoside analogs. It apparently requires the presence of both ribose-1-phosphate and free inorganic phosphate, and the latter does not inhibit the reaction up to concentrations of ca. 0.5 mM. The key question, whether this process involves repeated ribosylation-phosphorolysis cycle or perhaps some other activity, will be addressed in a separate paper.

## 3. Experimental

### 3.1. General

Recombinant *E. coli* and calf spleen PNP were obtained and purified as described elsewhere [31,32]. 2,6-Diamino-8-azapurine sulfate, N7-methylguanosine and 8-azaguanine were from Sigma-Aldrich (St. Louis, MO, USA.), the latter was re-crystallized as a monosodium salt. N7-methylguanosine was used without further purification. α-D-Ribose-1-phosphate was prepared enzymatically from N7-methylguanosine and inorganic phosphate, and assayed by previously described fluorimetric method [15]. The recombinant calf PNP was used as a catalyst, and the second reaction product, N7-methylguanine, was removed in nearly 97% by crystallization. All buffers were of analytical grade. Fluorescence was measured on a Varian Eclipse instrument (Varian Corp., Palo Alto, CA, USA), using 4-mm pathlength semi-micro cuvettes. Fluorescence quantum yields were determined either relative to tryptophan in water (ϕ = 0.15, [33]), or, in the case of the nucleosides, relative to DaaPur in water (ϕ = 0.40, [17]). The yields were determined with excitation at 280 nm, and are accurate to ±15%.

### 3.2. Enzymatic Reactions and Separation of the Products

Ribosylation of 2,6-diamino-8-azapurine and 8-azaguanine was carried out on a semi-micro scale, according to the following procedures:

(1) 5 mM DaaPur, diluted from the ammonium salt solution, in 25 mM HEPES, pH 6.6, reacted with ~7 mM ribose-1-phosphate in 1 mL volume, at 35 °C, for 3 h. Recombinant calf PNP (4 µL of 12.7 mg/mL solution) was used as a catalyst. Reaction progress was monitored fluorimetrically. After 3 h the reaction was stopped by boiling in a microwave oven for ~20 s and the resultant mixture was analyzed by HPLC (see Figure 4). The overall yields of N7-β-d- and N8-β-D-riboside were ca. 25% and 7%, respectively.
(2) To the same starting solution 0.5 mM inorganic phosphate was added. The reaction was allowed to run for 8 h at 32 °C. Analysis of the products (Figure 4) shown ca. 40% yield of the N8-β-d-riboside, with ~10% yield of the N7-β-d-riboside.
(3) Reaction was conducted for ~5 h under the conditions as indicated in (1) above, except that *E. coli* PNP was used as a catalyst. The resultant mixture contained N8-β-D-riboside (~30%, retention time ~17.5 min) and N9-β-d-riboside (~10%, 29–33 min).

Products were separated by reversed-phase HPLC (RP-HPLC) on a UFLC system from Shimadzu (Kyoto, Japan) equipped with UV (diode-array) detection at 280 nm and 315 nm. The column used was a Kromasil reversed-phase analytical C8 column (250 × 4.6 mm, 5-μm particle size). The eluent was deionized water (solvent A) and 80% methanol in water (solvent B). The solvent program was: 0–10 min, 100% solvent A (isocratic); 10–50 min, a linear gradient from 0 to 30% solvent B; 50–60 min, a linear gradient to 100% solvent B; 60–70 min, 100% solvent B.

## 4. Conclusions

We have shown that 2,6-diamino-8-azapurine undergoes enzymatic ribosylation by PNP in phosphate-free media, and the reaction products, N9-, N8- and N7-β-d-ribofuranosides, exhibit high fluorescence yields, which makes them applicable in analytical and mechanistic enzymology. With regards to fluorescence, the N8-riboside in particular seems to be an excellent indicator of the *E. coli* PNP in biological samples, while the N7-riboside is almost specific for the mammalian enzyme [16]. The assays based on these compounds may be important in view of the recent application of *E. coli* PNP in cancer gene therapy in the so-called suicidal gene strategy [34].

Various types of PNP may be used to selectively ribosylate the nucleobase analog in the desired location, and, as shown in the present paper, ribosylation sites are additionally dependent on the reaction conditions. Although the catalysis rates in this case are rather slow, we can expect that the application of various mutated forms of PNP, obtained via the site-directed mutagenesis [31,35], or enzymes from different sources [18,36] may lead to more efficient and more selective synthetic methods.
